# Relationship between early onset severe intrahepatic cholestasis of pregnancy and higher risk of meconium-stained fluid

**DOI:** 10.1371/journal.pone.0176504

**Published:** 2017-04-24

**Authors:** Maria C. Estiú, Maria A. Frailuna, Carla Otero, Marcela Dericco, Catherine Williamson, Jose J. G. Marin, Rocio I. R. Macias

**Affiliations:** 1 Ramón Sardá Mother’s and Children’s Hospital, Buenos Aires, Argentina; 2 Women's Health Academic Centre, King's College London, London, United Kingdom; 3 Experimental Hepatology and Drug Targeting (HEVEFARM), IBSAL, National Institute for the Study of Liver and Gastrointestinal Diseases (CIBERehd), University of Salamanca, Salamanca, Spain; Texas A&M University, UNITED STATES

## Abstract

**Background:**

Intrahepatic cholestasis of pregnancy (ICP) is the commonest gestational liver disease. The risk of adverse fetal outcome has been associated with the severity of maternal hypercholanemia after diagnosis.

**Objective:**

To investigate whether there is a relationship between the severity and timing of onset of hypercholanemia and the risk of meconium-stained amniotic fluid (MSAF) and adverse neonatal events.

**Study design:**

The study included 382 pregnancies complicated by ICP managed at a referral hospital in Buenos Aires (Argentina) between June 2009 and December 2013. The patients were classified into three groups according to the severity of hypercholanemia at diagnosis; mild (10–19.9 μmol/L), moderate (20–39.9 μmol/L) and severe (≥40 μmol/L). Their clinical characteristics and pregnancy outcomes were investigated in a prospective observational study.

**Results:**

Higher risk of MSAF was observed when ICP appeared early in gestation or when hypercholanemia was more severe. Taking both parameters into account an MSAF risk factor (MRF) was defined. Based on a model of positive/negative predictive values, a cut-off point of MRF = 3 was selected, which prioritized sensitivity *versus* specificity. In ICP patients with MRF>3, the probability of MSAF was enhanced 4-fold. An increase in the frequency of MSAF was also associated with higher serum levels at diagnosis of alanine transaminase, alkaline phosphatase and direct bilirubin.

**Conclusions:**

The risk of MSAF is associated not only with the magnitude of hypercholanemia at diagnosis but also with the early gestational onset of raised maternal serum bile acids.

## Introduction

Intrahepatic cholestasis of pregnancy (ICP) is usually diagnosed in the third trimester of gestation, although it may present as early as the first trimester. It typically resolves without treatment after delivery [1]. The etiopathogenesis of ICP involves genetic, hormonal and environmental factors [2], and diagnosis is based on the presence of pruritus and fasting elevated serum bile acid concentrations (≥10 μmol/L), i.e., hypercholanemia. ICP is not usually associated with immediate life-threatening complications for the mother, although recent studies have suggested that ICP patients have a significantly higher risk of developing gestational diabetes [3]. Also long term complications of ICP, such as hepatobiliary diseases, autoimmune-mediated diseases, cardiovascular problems and cancer have been reported [4,5].

As demonstrated in animal models, exposure to pathologically high levels of bile acids affects normal fetal development, in particular adversely impacting hepatobiliary function [6,7], with persistent abnormalities for several weeks after birth [8]. Ursodeoxycholic acid (UDCA) administration partially prevents the deleterious effects of maternal cholestasis on rat placenta [9,10], but has no effect on the altered expression of genes involved in neonatal hepatobiliary function [10].

The severity of hypercholanemia has been associated with higher risk of serious adverse events for the fetus, such as spontaneous and iatrogenic preterm delivery, fetal distress and intrauterine fetal death, all of which occur more frequently in pregnancies with higher peaks of maternal hypercholanemia [11–13]. High bile acid levels have also been associated with increased risk of MSAF [14]. This is very relevant because the exposure of fetal lung to toxic levels of these molecules can result in lung injury, through alteration of secretory phospholipase A2 [15,16].

The risk of perinatal mortality has been shown to be reduced by delivery at 36 weeks of gestation as compared with expectant management [17], although this study did not consider bile acid concentrations when deciding about timing of delivery [18]. Another study using a decision-analytic model to determine the optimal gestational age for delivery of women with ICP proposed that immediate delivery at 36 weeks of gestation without amniocentesis or corticosteroid administration reduced neonatal morbidity and mortality [19]. Early induction of labor at 37 weeks of gestation was found justified in high-risk ICP (≥40 μmol/L total bile acid concentration) and only a reduction in birth weight was found compared to women with lower serum bile acids [20]. A recent meta-analysis has shown that the close monitoring of ICP patients and treatment with UDCA are beneficial and account for a reduction in the rate of adverse pregnancy outcomes reported in the last years [21]. Furthermore, ICP is associated with long-term effects on the health of the offspring, including susceptibility to increased adiposity and metabolic disease [22].

Changes in serum levels of estrogens [23] and progesterone derivatives have been described in asymptomatic hypercholanemia of pregnancy [24] and ICP [25], but no clear association with the severity of hypercholanemia has been reported except for progesterone sulfates, which have been proposed as prognostic indicators for ICP [26].

The most popular way of establishing the severity of ICP is based on the magnitude of the highest peak of serum bile acid levels measured at any time after diagnosing ICP, usually late in the patient follow-up before delivery. A correlation between the degree of hypercholanemia and the occurrence of adverse fetal events was found [11–13].

However, by doing so, some important information has not been usually considered, such as i) the gestational age; ii) the degree of hypercholanemia at diagnosis, iii) whether the maternal serum levels of bile acids were corrected during gestation, and iv) the length of time the placenta and fetus are exposed to elevated bile acid levels in maternal blood.

The aim of the present study was to investigate whether there is a relationship between severity of hypercholanemia and the gestational week when it appears, and the risk of MSAF and adverse neonatal outcome in ICP pregnancies. We also evaluated whether treatment with UDCA reduces this risk.

## Material and methods

### Patient selection

This investigation was conducted using data collected prospectively from June 2009 to December 2013 at the Ramón Sardá Mother’s and Children’s Hospital in Buenos Aires (Argentina). Women with fasting hypercholanemia (serum bile acid levels ≥10 μmol/L) and pruritus of unexplained cause were invited to participate in the study. The exclusion criteria were: patients with any other hepatic disease or disease affecting liver tests, multiple-birth pregnancies or deliveries at another institution. Written informed consent from all patients was obtained. The study was reviewed and approved by the Medical Ethic Committee of the Ramón Sardá Mother's and Children's Hospital (2009/03/12).

### Definition of groups

According to the magnitude of hypercholanemia at diagnosis, which was measured with an enzymatic colorimetric method (Randox Laboratories, Crumlin, UK) [25], the patients were classified into three groups. In addition to the well-recognized level of severe ICP (≥40 μmol/L), two additional groups of mild (10–19.9 μmol/L) and moderate (20–39.9 μmol/L) ICP were defined for fine-tuning of the relationship between hypercholanemia and adverse outcome. Maternal age, parity, and the following adverse events were evaluated: stillbirth, perinatal death (between week 24 of gestation and 4 weeks after delivery), meconium aspiration syndrome (babies with infiltrates on chest X-ray), gestational age at delivery, preterm birth (<37 weeks of gestation, both spontaneous and elective), MSAF, placental abruption, asphyxia (arterial pH <7.05 and base deficit of >12 mmol/L or arterial pH <7.0), altered fetal vitality (5 min Apgar <7), fetal well-being (normal cardiotocograph trace), and intensive care unit admission. Information concerning the occurrence of diabetes, pre-eclampsia, gestational hypertension, anemia, and other pathologies was also retrieved from medical records.

To have a complete picture of the consequences of this pathology for the fetus, both severe and mild clinical alterations were included in the evaluation of the outcome of ICP pregnancies.

The infants that were small for gestational age (SGA) were defined as having a birth weight <10^th^ centile and those that were large for gestational age (LGA) as having birth weight >90^th^ centile, after correction for sex, parity and gestational age according to the Ramón Sardá Hospital growth charts. As part of the protocol of management of these patients in the hospital, maternal blood samples were collected twice per week from the time of diagnosis until delivery and two more samples were obtained 1–2 days and four weeks after birth.

Meconium risk factor (MRF) was defined as the ratio between serum bile acid concentrations (SBAd in μM) and the gestational age (GAd in weeks), both at the time of diagnosis, i.e, MRF = SBAd/GAd. A model of predicted positive and negative values was elaborated to select the cut-off value.

### Statistical analysis

Where the data are shown as means±SD, an ANOVA test was performed and the Bonferroni method of multiple-range testing was used to calculate the statistical significance of differences among groups. Categorical variables were compared with Chi-squared or Fisher’s exact tests. A probability of <0.05 was considered statistically significant. Stata software 11 (StataCorp, College Station, Texas, USA) was used for statistical analyses. The study is reported according to STROBE guidelines.

## Results

**Fig 1:**
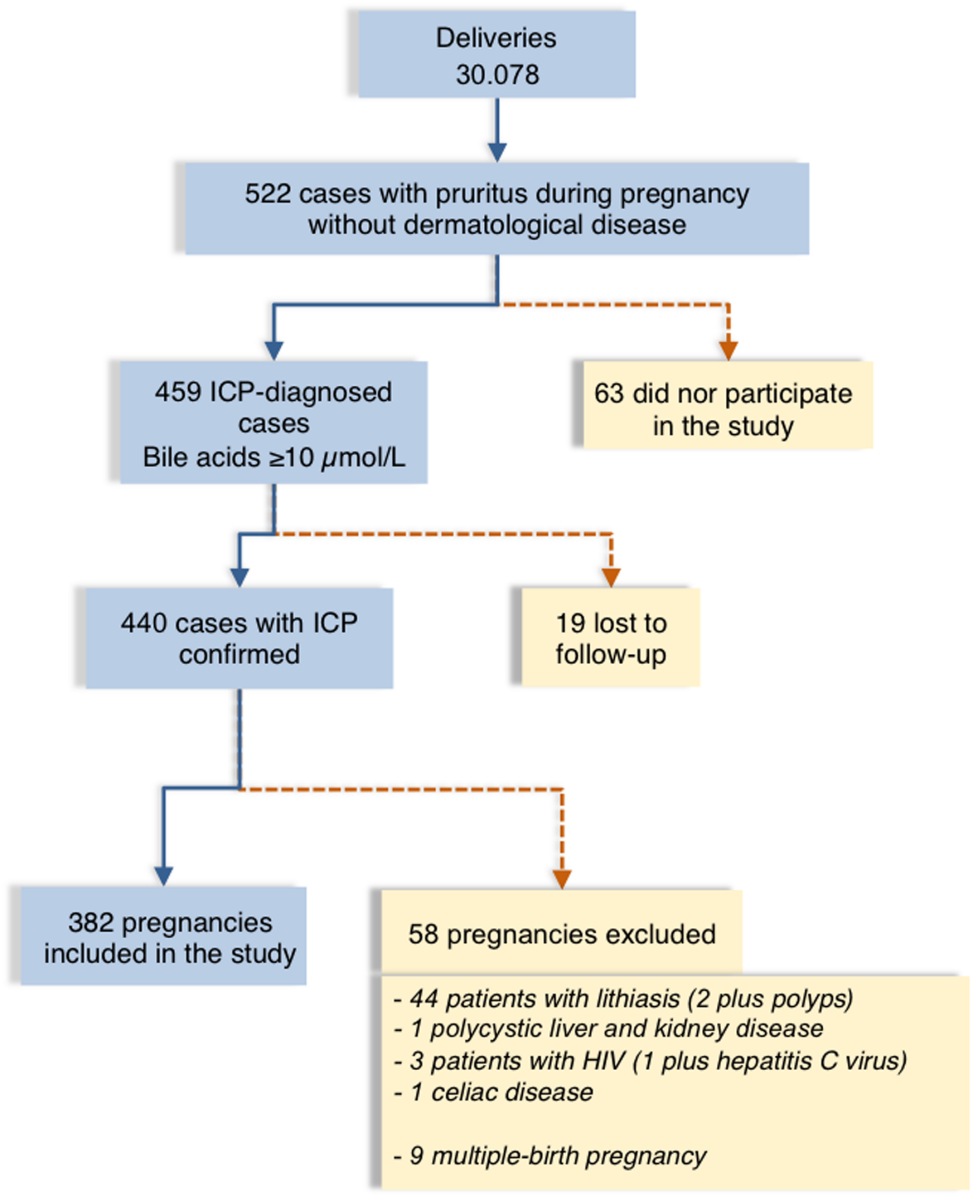
Flow chart of ICP cases included in the study. Flow chart showing the intrahepatic cholestasis of pregnancy (ICP) cases at the Mother’s and Children’s Hospital, Buenos Aires, from June 2009 to December 2013, and reasons for the exclusion of some patients from the study.

All pregnancies leading to delivery during the period of this study (n = 30,078) were screened for ICP if women reported pruritus. Of a total of 522 women with pruritus of not identified cause (Fig 1), 63 did not participate in the study, and 77 were excluded because their follow-up was interrupted (n = 19) or due to their particular characteristics other than ICP (n = 58) that could affect perinatal complications (Fig 1). Patients that completed the study (n = 382) were classified based on the mothers’ serum bile acid concentrations at diagnosis as having mild (n = 183), moderate (n = 115) or severe (n = 84) ICP. Table 1 shows the clinical and demographic characteristics of these patients.

**Table 1:** Demographic and clinical characteristics of patients.

Demographics	Intrahepatic cholestasis of pregnancy				
	Total (n = 382)	Mild (n = 183)	Moderate (n = 115)	Severe (n = 84)	p-value
Maternal age, y	26.0 ± 6.5	26.6 ± 6.5	25.8 ± 6.6	26.4 ± 6.2	ns
Gestational age at diagnosis, wk	33.6 ± 3.6	34.2 ± 3.4	33.5 ± 3.5	32.8 ± 4.1	ns
**Parity**, n (%)					
0	136 (35.6)	68 (37.2)	39 (33.9)	29 (34.5)	0.823
≥1	246 (64.4)	115 (62.8)	76 (66.1)	55 (65.5)	0.827
**Gestational age at diagnosis**, n (%)					
<24 wk	6 (1.6)	3 (1.6)	1 (0.9)	2 (2.4)	0.695
24 to <28 wk	25 (6.5)	9 (4.9)	8 (7.0)	8 (9.5)	0.360
28 to <32 wk	50 (13.1)	21 (11.5)	16 (13.9)	13 (15.5)	0.635
32 to <37 wk	229 (60.0)	108 (59.0)	68 (59.1)	53 (63.1)	0.800
≥37 wk	72 (18.8)	42 (23.0)	22 (19.1)	8 (9.5)	0.033
**Country of origin**, n (%)					
Argentina	229 (60)	114 (62.3)	67 (58.2)	48 (57.1)	0.660
Bolivia	75 (19.6)	28 (15.3)	29 (25.2)	18 (21.4)	0.099
Paraguay	58 (15.2)	30 (16.4)	14 (12.2)	14 (16.7)	0.560
Peru	14 (3.6)	8 (4.4)	4 (3.5)	2 (2.4)	0.718
Other	6 (1.6)	3 (1.6)	1 (0.9)	2 (2.4)	0.695
**Co-morbidities**, n (%)					
Threatened premature labor	6 (1.6)	0 (0)	4 (3.5)	2 (2.4)	0.050
Diabetes	31 (8.1)	18 (9.8)	9 (4.9)	4 (4.8)	0.367
Preeclampsia	7 (1.8)	6 (3.3)	0 (0)	1 (1.2)	0.107
Gestational hypertension	7 (1.8)	4 (2.2)	2 (0.9)	1 (1.2)	0.850
Hypothyroidism	5 (1.3)	2 (1.1)	2 (0.9)	1 (1.2)	0.887
Iron-deficiency anemia	31 (8.1)	14 (7.7)	6 (5.2)	11 (13.1)	0.126
Other medical conditions	7 (1.8)	1 (0.5)	3 (2.6)	3 (3.6)	0.175
**Pharmacological treatment**, n (%)					
Ursodeoxycholic acid	298 (78.0)	144 (78.7)	84 (73.0)	70 (83.3)	0.213
Dexchlorpheniramine	298 (78.0)	144 (78.7)	84 (73.0)	70 (83.3)	0.213
Ferrous sulfate	36 (9.4)	16 (8.7)	8 (7.0)	12 (14.3)	0.197
Folic acid	36 (9.4)	16 (8.7)	8 (7.0)	12 (14.3)	0.197
Alprazolan	1 (0.3)	1 (0.5)	0 (0)	0 (0)	0.580
Betametasone	120	76 (41.5)	39 (33.9)	5 (5.9)	0.000
Levotiroxine	5	2 (1.0)	2 (1.7)	1 (1.2)	0.887

Data are expressed as n (%), or mean ± SD. ns, p>0.05 comparing mild and moderate, mild and severe, and comparing moderate and severe. Other less frequent medical conditions include Hirschsprung's disease (mild, n = 1), syphilis (moderate, n = 2), dyslipidemia (moderate, n = 1), Chagas disease (severe, n = 1), irritable bowel syndrome (severe, n = 1) and syphilis (severe, n = 1). Definition of ICP groups according to serum bile acid concentrations: mild (10–19.9 μmol/L); moderate (20–39.9 μmol/L); severe (≥40 μmol/L).

Mean maternal age (26.0±6.5 years) was lower than in previous series [12,13], but similar to that of the general population of pregnant women attending this hospital. Mean gestational age at diagnosis was 33.6±3.6 weeks. Most cases were diagnosed between 32 and 37 weeks, although 18.8% were diagnosed later, and 21.3% earlier in pregnancy (Table 1). A trend for less severe hypercholanemia was found when the diagnosis was made closer to term.

ICP was diagnosed in one third of the patients in their first pregnancy (Table 1), while two-thirds of patients had at least one previous pregnancy lasting for more than 20 weeks. Analysis of the history of adverse events in these parous patients revealed frequent miscarriages (S1 Table) and gestational trophoblastic disease (one mole). In addition, 37.4% patients had a history of ICP, with 12.4% having had fetal death, and 28% preterm delivery (S1 Table). Interestingly, among women with very early diagnosis (<24 weeks), 67% had a history of ICP in previous gestations, and 33% had a history of stillbirth.

Maternal rates of co-morbidities were similar among all groups (Table 1). Five patients had hypothyroidism, but since no routine screening of thyroid disease in pregnant women is carried out at this hospital the data could underestimate the frequency of thyroid disease.

In recent years the number of immigrants from bordering countries has increased in Argentina and represented 43% of the total number of women attending the hospital between 2009 and 2013 (Table 1). The prevalence in the overall ICP population was ≈1.5%, with no differences in geographical origin.

S2 Table shows the complete profile of serum biochemical parameters evaluated at diagnosis. There was a significant increase in direct bilirubin in cases with more severe hypercholanemia, while no changes in total bilirubin, transaminases, γ-glutamyl-transpeptidase, alkaline phosphatase and total cholesterol, were found.

**Fig 2:**
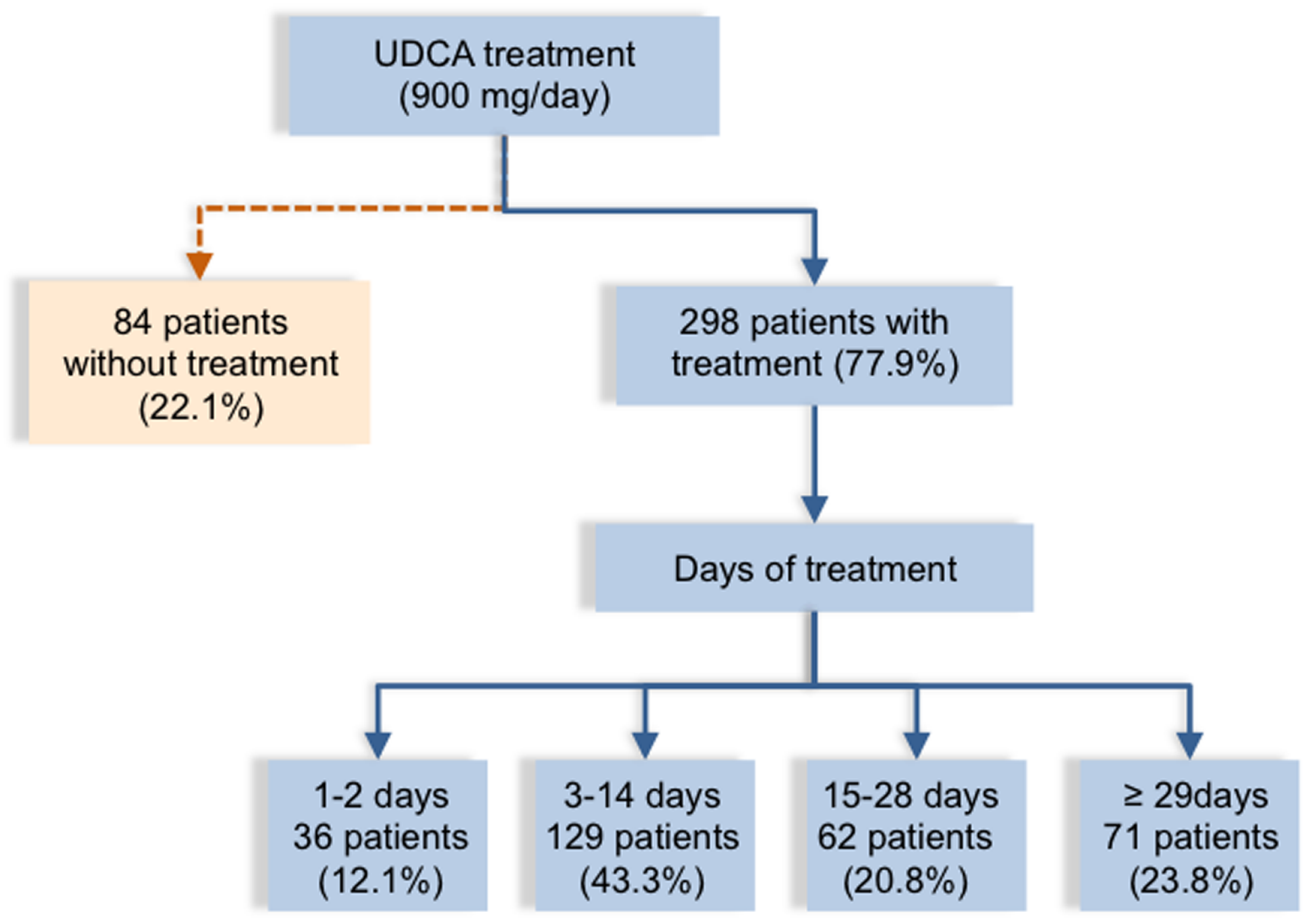
Flow chart of management of patients. Flow chart showing distribution of women with intrahepatic cholestasis of pregnancy (ICP) depending on whether they were not treated or treated with ursodeoxycholic acid (UDCA), together with days of treatment.

Most women diagnosed with ICP (77.9%) received treatment with UDCA (Table 1, Fig 2) at a dose of 300 mg three times a day plus the antihistamine dexchlorpheniramine to alleviate pruritus. The length of UDCA treatment varied from 1 to 150 days and started the day of diagnosis, even when elective delivery was chosen. Almost 50% were treated for <2 weeks (Fig 2), and only 20 for more than two months. The administration of iron supplement (ferrous sulfate) and levothyroxine corresponds to patients with anemia and hypothyroidism, respectively.

The obstetric outcome is shown in Table 2. Mean gestational age at delivery was similar in severe cases as compared to mild cases. In 353 neonates preterm birth and neonatal weight was categorized. Difference between gestational age calculated from the last menstrual period and the first ultrasound screening was lower than one week. Preterm births occurred in 225 ICP pregnancies (63.7%), much more frequently than in other recent studies [13,17]. Considering these preterm births, 16% were spontaneous and the frequency was similar in patients with different degrees of ICP severity. In 84% there was elective induction of labor due to ICP. Mode of delivery was determined according to the hospital Guideline for managments of ICP. In the general obstetric population at this hospital during the same period, elective preterm birth was 6-fold less frequent than in ICP women. There were two cases of placental abruption, both in women with severe ICP with diagnosis at 31 and 29 weeks of gestation.

**Table 2:** Comparison of obstetric outcomes.

Obstetric outcome	Intrahepatic cholestasis of pregnancy				
	Total (n = 382)	Mild (n = 183)	Moderate (n = 115)	Severe (n = 84)	p-value
**Gestational age at birth** (wk)	35.7 ± 1.8	36.1 ± 1.7	35.6 ± 1.8	35.0 ± 2.0	ns
**Birth weight** (g)	2815 ± 499	2896 ± 433	2821 ± 511	2631 ± 568	ns
**Neonatal sex** (male/female) (n/n)	198/184	92/91	66/49	40/44	0.333
**Mode of delivery**, n (%)					
Vaginal	158 (41.4)	80 (43.7)	47 (40.9)	31 (36.9)	0.572
Elective caesarean	224 (58.6)	103 (56.3)	68 (59.1)	53 (63.1)	0.572
**Perinatal outcome**, n (%)					
Meconium-stained amniotic fluid	48 (12.6)	15 (8.2)	13 (11.3)	20 (23.8)	0.0015
Meconium aspiration syndrome	0 (0)	0 (0)	0 (0)	0 (0)	-
Altered fetal heart rate	21 (5.5)	8 (4.4)	5 (4.4)	8 (9.5)	0.186
Placental abruption	2 (0.5)	0 (0)	0 (0)	2 (2.4)	0.028
Stillbirth	0 (0)	0 (0)	0 (0)	0 (0)	-
Perinatal death	1 (0.3)	0	0	1 (1.2)	0.169
Asphyxia	0 (0)	0 (0)	0 (0)	0 (0)	-
5 min Apgar <7	0 (0)	0 (0)	0 (0)	0 (0)	-
**Body weight/gestational age relationship**, n (%)*	(n = 353)*	(n = 168)	(n = 108)	(n = 77)	
Small weight for gestational age	6 (1.7)	2 (1.2)	2 (1.8)	2 (2.6)	0.724
Appropriate weight for gestational age	312 (88.4)	155 (92.3)	91 (84.3)	66 (85.7)	0.092
Large weight for gestational age	35 (9.9)	11 (6.5)	15 (13.9)	9 (11.7)	0.116
**Preterm birth <37 wk**, n (%)*	(n = 225)*	(n = 94)	(n = 70)	(n = 61)	
Spontaneous	36 (16.0)	16 (17.0)	10 (14.3)	10 (16.4)	0.890
Elective	189 (84.0)	78 (83.0)	60 (85.7)	51 (83.6)	0.890
Admission intensive care unit	36 (16.0)	14 (14.9)	12 (17.1)	10 (16.4)	0.922
Admission medium care	48 (21.3)	25 (26.6)	12 (17.1)	11 (18.0)	0.262
Respiratory distress syndrome	36 (16.0)	14 (14.9)	12 (17.1)	10 (16.4)	0.922

Data are expressed as n (%), or mean ± SD. ns, p>0.05 comparing mild and moderate, mild and severe, and moderate and severe. Definition of ICP groups according to serum bile acid concentrations: mild (10–19.9 μmol/L); moderate (20–39.9 μmol/L); severe (≥40 μmol/L).

*, 29 neonates were not included due to lack of accurate data on gestational age.

Fetal bradycardia was found in 28 cases without association with other complications. No cases of asphyxia or stillbirth were recorded and only one perinatal death occurred in the severe ICP group. The diagnosis of this latter case was made at 29 weeks of gestation with maternal serum bile acids of 83 μM. Spontaneous birth occurred 2 weeks after diagnosis, after placental abruption and detection of the presence of meconium-stained fluid. The Apgar scores of the baby girl (950 g) were 7 and 8 at 1 and 5 minutes, respectively. She had respiratory distress syndrome and a chromosomal abnormality (66,XXX).

**Fig 3:**
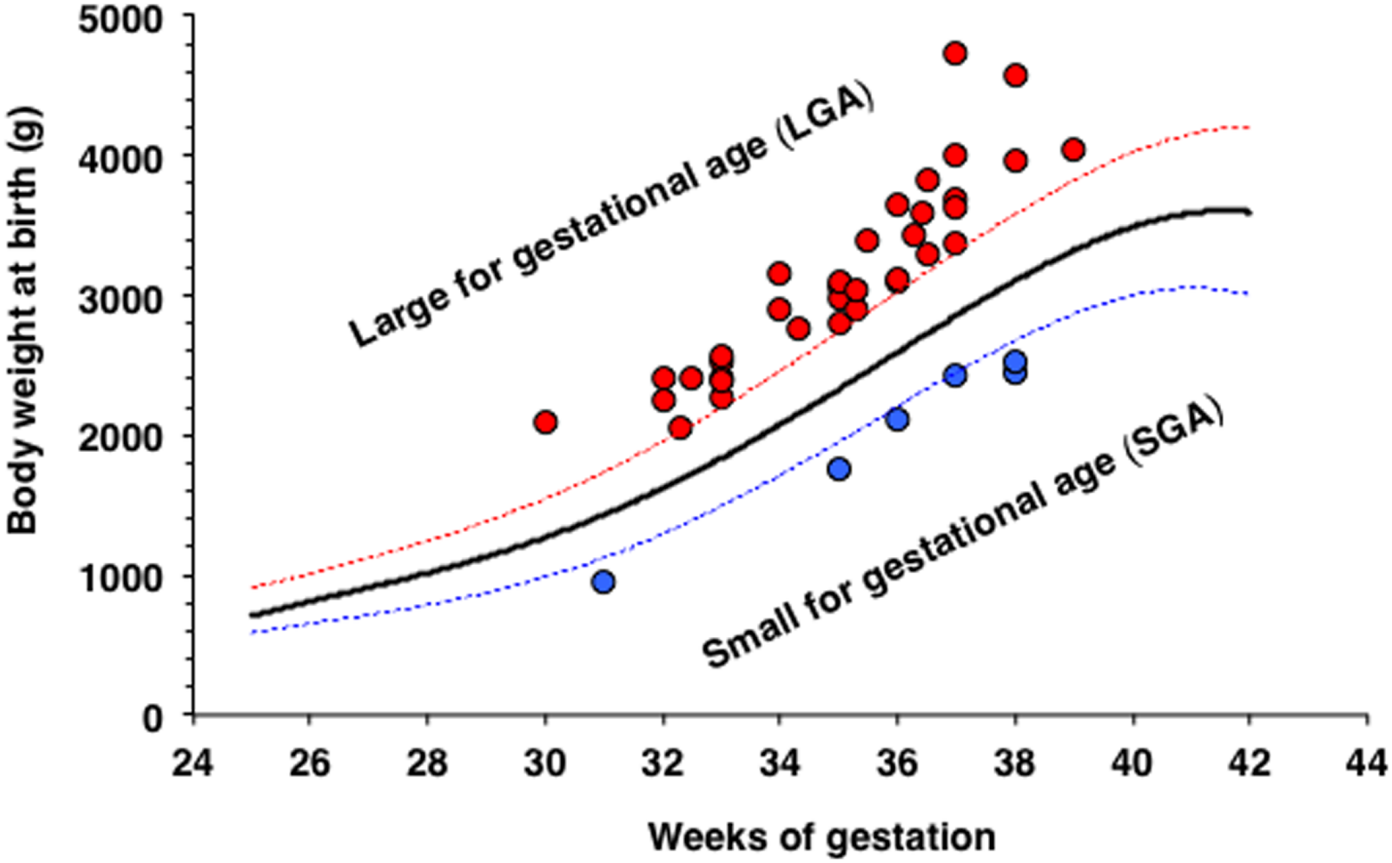
Relationship between hypercholanemia and body weight at birth. Gestational age and body weight of neonates born from women with intrahepatic cholestasis of pregnancy (ICP) with high or low weights at birth for their gestational age.

Once adjusted for gestational age, no significant differences in birth weight of the neonates, between ICP groups or with control neonates born in the hospital in the same period, were found. The birth weight of the neonates was classified as SGA and LGA (Table 2). In this study, 35 neonates (9.9%) were LGA (22 male/13 female); in 16 cases ICP was associated with other pathologies. The frequency of LGA cases was 4.4% in the general population of the hospital. Additionally, 6 neonates (1.7%) were SGA (2 male/4 female), four of them from women without other pathologies (Fig 3).

**Fig 4:**
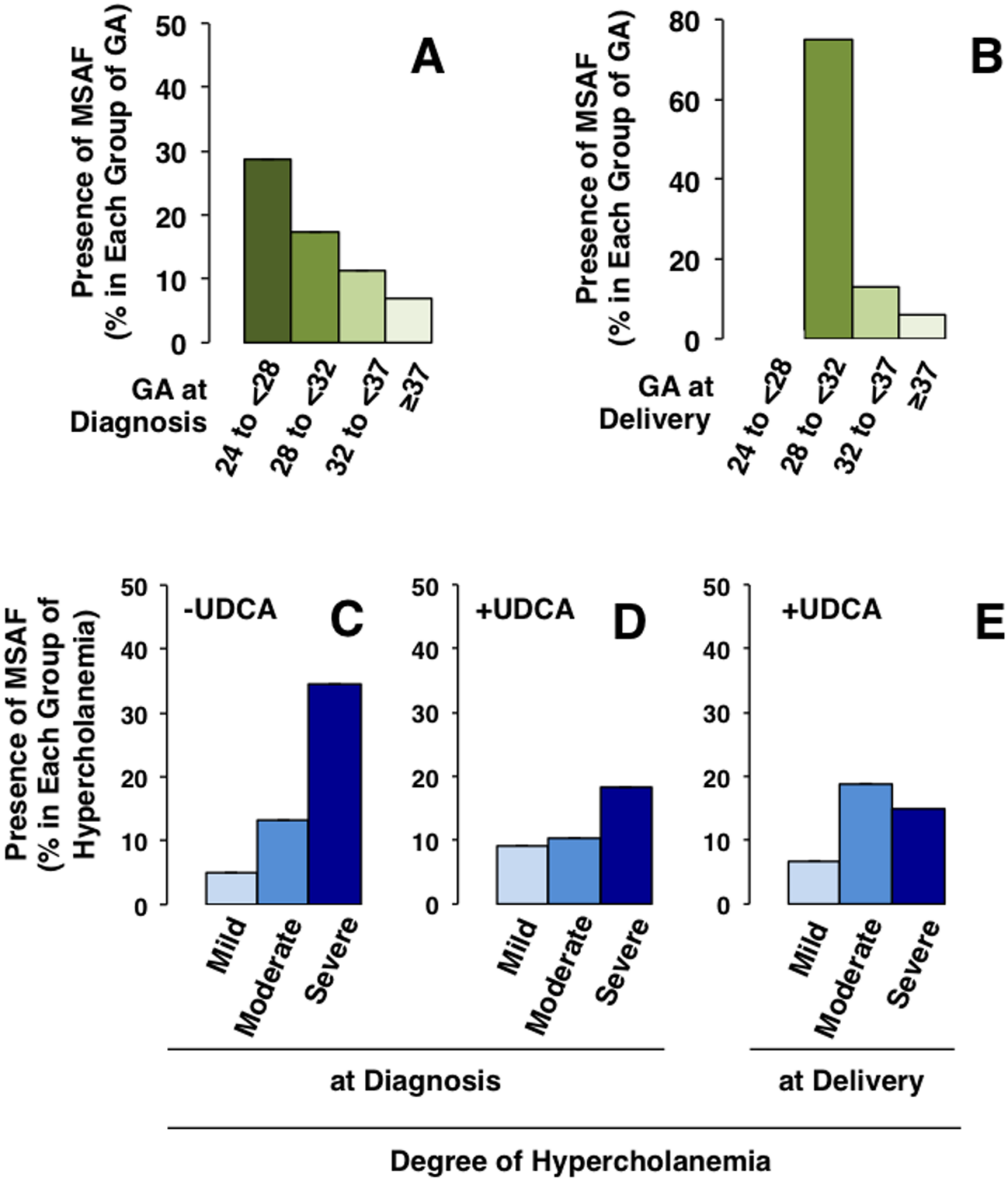
Relationship between hypercholanemia and the frequency of meconium-stained amniotic fluid (MSAF). Frequency of the presence of MSAF in pregnancies complicated by cholestasis of pregnancy classified according to gestational age (GA) at diagnosis (A) or at delivery (B), severity of hypercholanemia (mild: 10–19.9 μmol/L; moderate: 20–39.9 μmol/L; severe: ≥40 μmol/L) at diagnosis in women not treated (C) or women treated with ursodeoxycholic acid (UDCA), (D), and at delivery in those treated with UDCA (E).

Only 36/382 neonates (9.4%) were admitted to the intensive care unit due to respiratory problems, with a similar frequency in the three groups. Severe breathing problems (hyaline membrane disease) were observed in 5 of these babies. Another 48 neonates were sent to medium care unit, mainly due to their malnutritional status and hyperbilirubinemia. MSAF was found in 12.6% of all the deliveries (Table 2). This was diagnosed in half of the cases by amniocentesis, and no cases of meconium aspiration syndrome were detected. The frequency of MSAF was significantly higher (P = 0.019) when ICP was diagnosed at earlier gestational ages (Fig 4A) and when delivery occurred earlier (P<0.0001) (Fig 4B). MSAF was significantly more frequent when serum bile acid concentrations were higher at diagnosis (P = 0.0015). This was clearly seen in untreated pregnancies (Fig 4C), in which delivery occurred sooner (≤2 days) after diagnosis. The association between the severity of hypercholanemia at diagnosis (Fig 4C and 4D) or at birth (Fig 4E) and the presence of MSAF was modified by treatment with UDCA (Fig 4D and 4E). In the ICP patients, severe hypercholanemia (≥40 μmol/L), but also high levels of alanine transaminase (≥80 UI/L), alkaline phosphatase (≥900 UI/L) and direct bilirubin (≥0.3 mg/dL) were associated with MSAF in univariate analyses of maternal serum biochemical parameters at diagnosis, but not at delivery (S3 Table).

The rate of MSAF was significantly higher among women who underwent elective cesarean section. This was not surprising because in 79.2% of cases the antenatal detection of meconium in amniotic fluid obtained at amniocentesis was the reason to schedule operative delivery to prevent complications.

**Fig 5:**
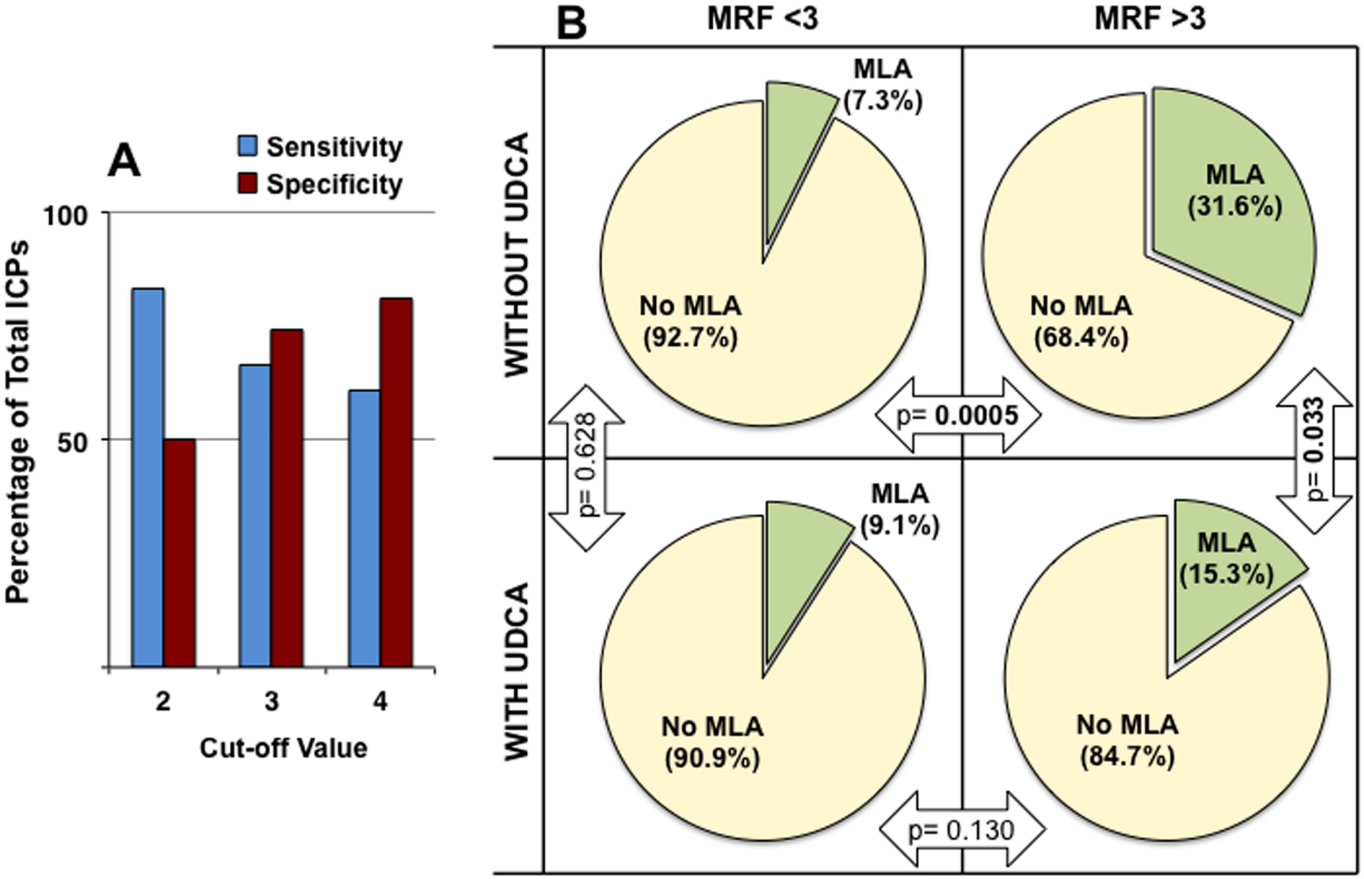
Evaluation of the index to predict the risk of meconium-stained amniotic fluid (MSAF). Balance between sensitivity and specificity for different cut-off values of meconium risk factor (MRF), calculated taking into account the severity of hypercholanemia and gestational age at diagnosis (A). Proportion of cases of MSAF in pregnancies with complications due to cholestasis of pregnancy according to a MRF cut-off value of 3 in women not treated or treated with ursodeoxycholic acid (UDCA) (B).

To evaluate the risk of MSAF in an attempt to improve the identification of patients with a higher risk of fetal complications, a new index was calculated (see “Material and methods” section). The MRF takes into account the two factors that were identified in the present study as being associated with higher frequency of MSAF, i.e., the degree of hypercholanemia (direct relationship) and gestational age at diagnosis (inverse relationship). Using a model of positive and negative predictive values, calculation of the sensitivity and specificity for different cut-off values of MRF was carried out (Fig 5A).

Using these criteria more useful balance between sensitivity (66.7%) and specificity (74.5%) was obtained for MRF = 3. In untreated ICP patients MSAF frequency was significantly lower (7.3%) in MRF negative cases than in MRF positive gestations (31.6%) (Fig 5B). Moreover, in the MRF positive group treatment with UDCA significantly reduced the risk of MSAF (Fig 5B).

## Discussion

To our knowledge, the present study is the largest one carried out on a Latin American population. In our series of pregnant women, the incidence of ICP was close to 1.5%. This is higher than the incidence of 0.2–0.7% found for ICP in most European and North American series [14,27], and lower than the reported incidence of ICP in Bolivia and Chile of 15% in older studies [28]. However, more recent studies in the same geographical area have reported a lower incidence [29], consistent with our study.

The aim of the management of women with ICP is to reduce discomfort from pruritus and to prevent adverse fetal/neonatal outcomes for which there are currently no reliable predictive tests. In borderline cases, clinicians make their decisions taking into account the gestational age and the evolution of ICP-associated hypercholanemia. Liver function test based on serum biochemistry profile and bile acid determinations are typically performed every 3–7 days. Many clinicians also elect to perform intrapartum fetal heart rate monitoring, although this procedure provides limited additional information to predict the outcome.

The peak value of hypercholanemia determined during the follow-up of these patients has been commonly used to establish the severity of ICP. Because this condition is characterized by dynamic changes in maternal serum bile acid levels the management of these patients is usually adapted over time. As adverse pregnancy outcomes do not occur in pregnancies where the maternal serum bile acid concentration is <40 μmol/L [11], expectant management may be considered for women with persistent mild hypercholanemia, i.e., from ≥10 μmol/L to <40 μmol/L serum bile acid concentrations. These women are commonly treated with UDCA, which improves pruritus and serum biochemistry, including serum bile acid levels in an important proportion of patients [30,31]. Only about a quarter of patients do not respond to this treatment [25].

The induction of labor before the 37th week of gestation, once fetal lung maturity has been verified and sometimes after antenatal administration of corticosteroids to accelerate lung maturity, is the standard protocol used at many hospitals with the aim of preventing stillbirth [11], although this strategy increases the risk of preterm-associated complications. Compared with expectant management, immediate delivery at 36 weeks of gestation has been reported to reduce perinatal complications in ICP [17,19]. In the present series, the lack of cases of stillbirth from 225 women whose babies were delivered before 37 weeks of gestation supports the beneficial effect of this strategy.

Changes in the management and close monitoring of ICP patients have reduced the frequency of stillbirths from rates of 7%, some years ago [32,33] to 3.5% or less in more recent studies [13,14,33], however, the risk of stillbirth and other less severe perinatal complications, such as MSAF, justify the search for prognostic factors that can help clinicians to choose the best option for each patient at each moment.

MSAF is a sign of fetal distress whose detection indicates the need for expedited delivery to prevent babies from developing meconium aspiration syndrome if they inhale meconium-stained fluid. This is a very rare complication, but it can be fatal and when it appears in preterm infants it is associated with higher neonatal morbidity [34]. In the present series, these neonates were treated in intensive care units with close monitoring and all of them had good outcomes.

In control pregnancies from this hospital and also in other studies [12,35], MSAF appears more frequently in births close to term and especially in post-date deliveries; this has been associated with a more advanced maturation of the gastrointestinal tract.

This study supports previous research demonstrating an association between MSAF and lower gestational age both at diagnosis and at birth in ICP. The strongest association with the occurrence of MSAF is found with higher degree of hypercholanemia at diagnosis. UDCA treatment reduces the risk of MSAF. Moreover, the present study also supports results from a recent study on a smaller number of ICP patients (59 women), which suggested that gestational age at diagnosis and serum bile acid levels are associated with adverse perinatal respiratory problems [36].

The mechanism by which high bile acid levels could cause MSAF is not clear but it has been proposed that it could be due to stimulation of colonic motility or be a result of fetal distress itself [37,38].

The presence of bile acids in meconium can result from bile reaching the fetal duodenum and/or from the amniotic fluid ingested by the fetus. A recent study analyzing bile acid species in the biliary tract and in the intestinal meconium of infants who died from miscarriage or soon after birth reported the presence of sulfate-conjugated bile acids in the meconium but not in gallbladder bile, which suggests that their origin probably is mainly the amniotic fluid swallowed by the fetuses [39]. Bile acid concentrations in amniotic fluid are 70-fold higher in ICP pregnancies than in controls [40], and are also significantly higher in the meconium of newborns from women with ICP than in controls [41]. This supports that enhanced colonic motility could be involved in MSAF.

An easy and accurate method for calculating the degree of the risk of MSAF would be a useful tool to help the obstetrician to take decisions regarding the management of ICP patients based on the probability of facing fetal and neonatal complications. Consequently, cases with moderate hypercholanemia but early onset of ICP must be closely monitored. The proposed MRF is a novel index that has proved to be reasonably useful for its incorporation into the follow up of these pregnancies. Moreover, the result from calculating MRF reflects the beneficial effect on the fetus of treating ICP patients with UDCA. However, the actual accuracy of MRF needs to be confirmed in further prospective studies.

## Supporting information

S1 Table History of adverse events in ICP patients with previous gestations >20 weeks. (DOC)

S2 Table Maternal serum biochemistry at diagnosis. (DOC)

S3 Table Univariate analyses of maternal serum biochemical parameters at diagnosis and at delivery for meconium staining amniotic fluid (MSAF) in patients with intrahepatic cholestasis of pregnancy. (DOC)
