# Supplementary material for: Relationship between early onset severe intrahepatic cholestasis of pregnancy and higher risk of meconium-stained fluid
Source: PLoS One. 2017 Apr 24;12(4):e0176504. doi: 10.1371/journal.pone.0176504 (PMC5402936; doi:10.1371/journal.pone.0176504)
Supplement: S1 Table — (DOC) [file pone.0176504.s001.doc]

| **History of** | **Intrahepatic cholestasis of pregnancy** | | | | |
| --- | --- | --- | --- | --- | --- |
|  | **Total** (n=214) | **Mild**  (n=104) | **Moderate** (n=61) | **Severe** (n=49) | **p-value** |
| Cholestasis of pregnancy, n (%) | 80 (37.4) | 38 (36.5) | 18 (29.5) | 24 (49.0) | 0.107 |
| Fetal death, n (%) | 27 (12.6) | 13 (12.5) | 7 (11.5) | 7 (14.3) | 0.906 |
| Preterm delivery, n (%) | 60 (28.0) | 22 (21.1) | 20 (32.8) | 18 (36.7) | 0.084 |
| Abortions, n (%) | 57 (26.6) | 24 (23.1) | 18 (29.5) | 15 (30.6) | 0.515 |
| Gestational trophoblastic disease, n (%) | 1 (0.5) | 1 (1.0) | 0 (0) | 0 (0) | 0.588 |

**S1 Table**. History of adverse events in ICP patients with previous gestations >20 weeks.

Definition of ICP groups according to serum bile acid concentrations: mild (10-19.9 µmol/L); moderate (20-39.9 µmol/L); severe (≥40 µmol/L).
