# Supplementary material for: Relationship between early onset severe intrahepatic cholestasis of pregnancy and higher risk of meconium-stained fluid
Source: PLoS One. 2017 Apr 24;12(4):e0176504. doi: 10.1371/journal.pone.0176504 (PMC5402936; doi:10.1371/journal.pone.0176504)
Supplement: S2 Table — (DOC) [file pone.0176504.s002.doc]

| **Biochemical tests** | **Intrahepatic cholestasis of pregnancy** | | | | |  |
| --- | --- | --- | --- | --- | --- | --- |
|  | **Total** (n=382) | **Mild**  (n=183) | **Moderate**  (n=115) | **Severe** (n=84) | **p-value** | **Reference ranges** |
| Bile acids (µmol/L) | 31.1 ± 27.1 | 14.7 ± 2.5 | 27.1 ± 5.7 | 72.6 ± 31.1 | a,b,c | <10 μmol/L |
| Alanine transaminase (IU/L) | 110 ± 99 | 88 ± 81 | 121 ± 139 | 145 ± 146 | ns | 4-36 IU/L |
| Aspartate transaminase (IU/L) | 71 ± 64 | 59 ± 43 | 74 ± 69 | 93.3 ± 85 | ns | 8-33 IU/L |
| γ-glutamyl transpeptidase (IU/L) | 23.0 ± 14.4 | 22.6 ± 16.2 | 23.1 ± 15.0 | 23.8 ± 11.9 | ns | 150-450 IU/L |
| Alkaline phosphatase (IU/L) | 577 ± 215 | 582 ± 209 | 567 ± 222 | 585 ± 207 | ns | <38 IU/L |
| Total bilirubin (mg/dL) | 0.82 ± 0.80 | 0.76 ± 0.27 | 0.81 ± 0.32 | 0.95 ± 0.46 | ns | <1.2 mg/dL |
| Direct bilirubin (mg/dL) | 0.27 ± 0.29 | 0.21 ± 0.24 | 0.25 ± 0.21 | 0.42 ± 0.37 | b,c | <0.25 mg/dL |
| Cholesterol (mg/dL) | 295 ± 64 | 289 ± 61 | 299 ± 61 | 305 ± 67 | ns | <300 mg/dL |

**S2 Table**. Maternal serum biochemistry at diagnosis.

Values are expressed as means ± SD. **^a^**, p<0.05 comparing mild and moderate; **^b^**, p<0.05 comparing mild and severe; **^c^**, p<0.05 comparing moderate and severe; ns, p>0.05. Reference ranges correspond to parameters in healthy pregnant women. Definition of ICP groups according to serum bile acid concentrations: mild (10-19.9 µmol/L); moderate (20-39.9 µmol/L); severe (≥40 µmol/L).
