# Supplementary material for: Relationship between early onset severe intrahepatic cholestasis of pregnancy and higher risk of meconium-stained fluid
Source: PLoS One. 2017 Apr 24;12(4):e0176504. doi: 10.1371/journal.pone.0176504 (PMC5402936; doi:10.1371/journal.pone.0176504)
Supplement: S3 Table — (DOC) [file pone.0176504.s003.doc]

| **Biochemical parameters** | | **At diagnosis** | | | **At delivery** | | |
| --- | --- | --- | --- | --- | --- | --- | --- |
| **MSAF** | | **Yes** | **No** | **P** | **Yes** | **No** | **P** |
| Bile acids | ≥ 40 µmol/L | 20 | 64 | **0.000** | 6 | 34 | 0.444 |
|  | < 40 µmol/L | 28 | 270 |  | 24 | 198 |  |
| Alanine transaminase | ≥ 80 IU/L | 28 | 143 | **0.043** | 15 | 78 | 0.078 |
|  | < 80 IU/L | 20 | 191 |  | 15 | 154 |  |
| Aspartate transaminase | ≥ 80 IU/L | 17 | 84 | 0.131 | 10 | 36 | **0.015** |
|  | < 80 IU/L | 31 | 250 |  | 20 | 197 |  |
| Alkaline phosphatase | ≥ 900 IU/L | 7 | 19 | **0.022** | 1 | 7 | 0.925 |
|  | < 900 IU/L | 41 | 315 |  | 29 | 225 |  |
| γ-glutamyl transpeptidase | ≥ 40 IU/L | 9 | 32 | 0.055 | 3 | 24 | 0.953 |
|  | < 40 IU/L | 39 | 302 |  | 27 | 208 |  |
| Total bilirubin | ≥ 1.3 mg/dL | 7 | 24 | 0.079 | 0 | 7 | 1.00 |
|  | < 1.3 mg/dL | 41 | 310 |  | 30 | 225 |  |
| Direct bilirubin | ≥ 0.3 mg/dL | 21 | 83 | **0.006** | 7 | 33 | 0.186 |
|  | < 0.3 mg/dL | 27 | 251 |  | 23 | 199 |  |

**S3 Table**. Univariate analyses of maternal serum biochemical parameters at diagnosis and at delivery for meconium staining amniotic fluid (MSAF) in patients with intrahepatic cholestasis of pregnancy.
